# Single-nucleotide polymorphisms and the effectiveness of taxane-based chemotherapy in premenopausal breast cancer: a population-based cohort study in Denmark

**DOI:** 10.1007/s10549-022-06596-2

**Published:** 2022-04-30

**Authors:** Cathrine F. Hjorth, Per Damkier, Tore B. Stage, Søren Feddersen, Stephen Hamilton-Dutoit, Mikael Rørth, Bent Ejlertsen, Timothy L. Lash, Thomas P. Ahern, Henrik T. Sørensen, Deirdre Cronin-Fenton

**Affiliations:** 1 Department of Clinical Medicine and Department of Clinical Epidemiology, Aarhus University Hospital, Aarhus University, Olof Palmes Allé 43-45, 8200 Aarhus N, Denmark; 2 Department of Clinical Pharmacology, Odense University Hospital, Odense, Denmark; 3 Department of Clinical Research, University of Southern Denmark, Odense, Denmark; 4 Department of Public Health, University of Southern Denmark, Odense, Denmark; 5 Clinical Pharmacology, Pharmacy and Environmental Medicine, Department of Public Health, University of Southern Denmark Odense, Odense, Denmark; 6 Department of Clinical Biochemistry, Odense University Hospital, Odense, Denmark; 7 Department of Pathology, Aarhus University Hospital, Aarhus N, Denmark; 8 Department of Oncology, Rigshospitalet, Copenhagen University, Copenhagen, Denmark; 9 Danish Breast Cancer Group, Rigshospitalet, Copenhagen University Hospital, Copenhagen, Denmark; 10 Department of Epidemiology, Rollins School of Public Health, Emory University, Atlanta, GA USA; 11 Department of Surgery, The Robert Larner, M.D. College of Medicine, The University of Vermont, Burlington, VT USA

**Keywords:** Breast neoplasm, Taxanes, Single-nucleotide polymorphisms, Mortality, Neoplasm recurrence

## Abstract

**Purpose:**

Taxane-based chemotherapy is the primary treatment for premenopausal breast cancer. Although being inconsistent, research suggests that variant alleles alter pharmacokinetics through reduced function of OATP transporters (limiting hepatic uptake), CYP-450 enzymes (hampering drug metabolism), and ABC transporters (decreasing clearance). Reduced function of DNA repair enzymes may hamper effectiveness through dose-limiting toxicities. We investigated whether single-nucleotide polymorphisms (SNPs) were associated with breast cancer recurrence or mortality in premenopausal women diagnosed with breast cancer.

**Methods:**

We conducted a population-based cohort study of premenopausal women diagnosed with non-distant metastatic breast cancer in Denmark during 2007‒2011, when guidelines recommended adjuvant combination chemotherapy (taxanes, anthracyclines, and cyclophosphamide). Using archived formalin-fixed paraffin-embedded primary tumor tissue, we genotyped 26 SNPs using TaqMan assays. Danish health registries provided data on breast cancer recurrence (through September 25, 2017) and death (through December 31, 2019). We fit Cox regression models to calculate crude hazard ratios (HRs) and 95% confidence intervals (CIs) for recurrence and mortality across genotypes.

**Results:**

Among 2,262 women, 249 experienced recurrence (cumulative incidence: 13%) and 259 died (cumulative incidence: 16%) during follow-up (median 7.0 and 10.1 years, respectively). Mortality was increased in variant carriers of *GSTP1* rs1138272 (HR: 1.30, 95% CI 0.95–1.78) and *CYP3A* rs10273424 (HR: 1.33, 95% CI 0.98–1.81). *SLCO1B1* rs2306283 (encoding OATP1B1) variant carriers had decreased recurrence (HR: 0.82, 95% CI 0.64–1.07) and mortality (HR: 0.77, 95% CI 0.60–0.98).

**Conclusion:**

Docetaxel effectiveness was influenced by SNPs in *GSTP1, CYP3A,* and *SLCO1B1* in premenopausal women with non-distant metastatic breast cancer, likely related to altered docetaxel pharmacokinetics. These SNPs may help determine individual benefit from taxane-based chemotherapy.

**Supplementary Information:**

The online version contains supplementary material available at 10.1007/s10549-022-06596-2.

## Background

In 2020, more than 2.3 million women were diagnosed with breast cancer worldwide, making female breast cancer the most frequent non-skin malignancy in women [1]. About one-third of breast cancer diagnoses occur in premenopausal women [2]. These women are usually recommended taxane-based chemotherapy as a primary treatment. Advances in breast cancer diagnosis and increasingly effective treatments (including the introduction of taxanes) have enlarged the pool of breast cancer survivors [3–5]. Still, mortality measured up to 15 years after premenopausal breast cancer ranges from 11 to 14% in high-income countries [2, 6]. Reasons for variation in individual treatment effectiveness are likely multifactorial. Studies suggest that taxane effectiveness may be up- or down-regulated by inherited single-nucleotide polymorphisms (SNPs) in genes involved in taxane transport and metabolism [7, 8].

The metabolism of taxanes—docetaxel and paclitaxel—occurs primarily in the liver. Taxane metabolites are eliminated through the bile. Solute carrier anion transporters (mainly OATP1B1, encoded by the polymorphic *SLCO1B1*) transport taxanes into hepatocytes, where they are metabolized by cytochrome P450 (CYP) enzymes [9]. Our study focused only on docetaxel, which is mainly metabolized by CYP3A4 (encoded by *CYP3A4*) and CYP3A5 (encoded by *CYP3A5*) into its main metabolites and conjugated by glutathione-*S*-transferase P1 (encoded by *GSTP1)*. Each of these enzymes is encoded by polymorphic genes. Taxanes are excreted into bile by efflux proteins encoded by the ATP-binding cassette (ABC) transporters. For docetaxel, this is mainly done by proteins encoded by the polymorphic genes *ABCB1*, *ABCC2, ABCG2,* and *ABCC1* [10]. SNPs in DNA repair genes, e.g., Eph-receptor A *(EPHA)* and excision repair cross-complementing genes (*ERCC),* may be associated with taxane toxicities [11], potentially leading to treatment discontinuation and reduced treatment effectiveness.

Research suggests that clinical outcomes (survival, progression-free survival, and tumor response) in cancer patients treated with taxanes may depend on genetic differences in taxane transporters [12, 13] and/or metabolizing enzymes [13, 14]. However, findings are rarely replicated [15, 16] and available studies have several limitations—including small sample size, population stratification, and different treatment schedules. As such, it is not possible to determine the impact of these genetic differences on taxane effectiveness from the existing literature.

We therefore investigated the association of SNPs that may influence taxane metabolism and transport with breast cancer recurrence and mortality in a large population-based cohort of premenopausal women diagnosed with non-distant metastatic breast cancer treated with docetaxel-based adjuvant chemotherapy.

## Materials and methods

### Setting and design

We conducted this nationwide population-based cohort study in Denmark. Denmark has a free tax-supported health care system [17]. The civil personal registration number, assigned to all residents upon birth or immigration, allows individual-level data linkage across Danish administrative and health registries [17]. In Denmark, all diagnostic surgical and biopsy tissue specimens are stored permanently as primary formalin-fixed paraffin-embedded (FFPE) tissue blocks at local pathology departments and registered in the Danish National Pathology Registry [18]. The Danish Breast Cancer Group (DBCG) is responsible for clinical guidelines on breast cancer diagnosis and treatment in Denmark. The DBCG clinical database records clinical and follow-up data for all Danish patients with invasive breast tumors [19], including data for up to 10 years of active follow-up for recurrence [20]. The DBCG registers breast cancer patients through an electronic reporting system accessible to all Danish pathology departments. This database is supplemented with data from other medical registries (e.g., the Danish National Pathology Registry). Subsequent clinical data from follow-up examinations also are added to the database. Until 2016, women diagnosed with breast cancer in Denmark were followed up first with semi-annual clinical exams for five years and then with annual exams during the following five years. Since 2016, patients have been able to choose among patient-led, nurse-led, or fixed annual follow-up exams. All women are offered mammography, ultrasound screening, and open access to a breast cancer unit for 10 years after a breast cancer diagnosis [21].

### Study cohort

We nested our study in the Predictors of Breast Cancer Recurrence (ProBe CaRe) cohort, which is described in greater detail elsewhere [22]. Briefly, the ProBe CaRe cohort includes premenopausal Danish women diagnosed with incident non-distant metastatic breast cancer during 2002–2011. We restricted the cohort to women diagnosed with breast cancer after implementation of docetaxel in combination with cyclophosphamide and sometimes epirubicin as guideline chemotherapy in Denmark (January 01, 2007). The cohort was further restricted to patients who were 18–55 years at diagnosis, who received chemotherapy, and who had FFPE blocks available in the pathology archives. Finally, we excluded women who experienced a recurrence or were lost to follow-up during the first six months after diagnosis. The study flowchart is provided in the Supplementary material (Fig. S1).

### Tissue procurement, DNA extraction, and genotyping

Procedures for collection of FFPE tumor blocks, preparation of the tumor tissue, and DNA extraction are described in detail elsewhere [23]. Based on a comprehensive review of the taxane pharmacogenetic literature and underlying biology, we identified 26 candidate SNPs. SNPs were considered candidates if (1) located in genes encoding proteins involved in taxane metabolism or transport, (2) previous studies suggested their biologically plausible role in taxane pharmacokinetics or effectiveness, or (3) they were associated with taxane toxicities (primarily neuropathy). A list of genotyped variants is presented in Table 2. Seven of these SNPs had been genotyped previously, as described elsewhere [23]. The remaining 19 SNPs were genotyped using commercially available TaqMan assays on a StepOne Plus real-time instrument (Applied Biosystems, Thermo Fisher Scientific, Foster City, California, USA) in accordance with the manufacturer’s protocol. For TaqMan assays, 20 ng of purified DNA extracted from FFPE tissues were amplified in 10µL PCR. The PCR were incubated at 60 °C for 30 s and 95 °C for 10 min and then cycled 50 times between 15-s incubations at 95 °C and 60-s incubations at 60 °C. Genotypes were classified based on TaqMan VIC/FAM intensity values using the auto-call feature of QuantStudio Software V1.3. Genotype results were manually inspected, and acceptance was overridden manually if irregular amplification curves were observed. We calculated expected genotype frequencies under Hardy–Weinberg Equilibrium (HWE) and compared them with observed frequencies both visually and by applying a traditional test for HWE. In addition, we compared allele frequencies between the study cohort and benchmarks reported for female European non-Finnish cohorts in the Genome Aggregation Database (gnomAD) [24]. For each SNP, we primarily classified each woman as (1) wildtype when she carried two normal alleles, (2) variant carriers (including both hetero- and homozygotes), or (3) as heterozygote or homozygote variant carriers.

### Data collection from Danish health registries

From the DBCG clinical database, we collected information on patient age at diagnosis, tumor characteristics (hormone receptor status, pathological grade, number of positive lymph nodes, and tumor size), cancer treatment (surgery type, intention-to-treat radiotherapy, adjuvant chemotherapy, and endocrine therapy), and dates of recurrences or second primary malignancies. From the Cause of Death Registry, we collected dates and causes of death, and from the Danish National Patient Registry we collected data on comorbid conditions diagnosed up to 10 years before the breast cancer diagnosis [25, 26].

### Outcomes

We used breast cancer recurrence and all-cause mortality as outcomes of taxane effectiveness. We adopted the DBCG definition of recurrence, which encompasses locoregional recurrence (tumor growth in the surgical scar, the ipsilateral breast, or regional lymph nodes), distant recurrence, or contralateral breast cancers diagnosed up to 10 years after initial breast cancer treatment [20]. Diagnosis of recurrence is either based on clinical assessment, pathological assessment, imaging, or a combination of these. We examined all-cause mortality, assuming breast cancer to be the most likely underlying or contributing cause of death in our young study cohort. We also examined breast cancer-specific mortality (BCSM) in sensitivity analyses, defined by deaths with breast cancer (ICD-10: C50) as the underlying or contributory cause of death.

### Other covariates

Comorbidities were summarized using the Charlson Comorbidity Index [27] and categorized as none, 1–2, or  ≥ 3 comorbidities (Supplemental Table S1). We derived cancer stage (categorized as stage I–III according to the TNM staging system [28]). Estrogen receptor (ER) status was a composite variable incorporating both negative and positive ER statuses and receipt of endocrine therapy. Grades 1–3 were assigned to lobular and ductal tumors; other tumors were not graded. Data on treatments included surgical procedure and intention-to-treat radiotherapy. Breast cancers were considered triple negative if tumors were ER–, human epidermal growth factor receptor 2 was negative, and information on progesterone receptor was either negative or missing.

### Statistical analyses

We started follow-up six months after the date of breast cancer surgery (date of diagnosis), to approximate the end of chemotherapy treatment. In analyses of recurrence, we censored follow-up upon death, emigration, diagnosis with new primary malignancy, last visit if lost to follow-up exams, end of the DBCG follow-up protocol (maximum 10 years), or end of available data (September 25, 2017). When examining mortality, longer available follow-up time in the Cause of Death registry allowed us to extend the follow-up period. Thus, mortality follow-up continued until the date of death, emigration, or end of data availability on December 31, 2019. We computed cumulative incidences of recurrence and death, considering death as a competing risk when examining recurrence. We used cause-specific Cox regression models to compute unadjusted hazard ratios (HRs) of recurrence and mortality for each SNP.

We also stratified all models by ER status and stage to evaluate effect measure modification by these factors. To account for potential underreporting of recurrence [29], we performed a sensitivity analysis considering recurrences to include BCSM in women not registered with a recurrence. To test the robustness of our mortality model, we restricted an analysis to BCSM. We used SAS 9.4 for all analyses (Cary, NC).

## Results

A total of 2,979 women were diagnosed during the 2007–2011 period in the ProBe CaRe cohort. The final study cohort included 2,262 women (Supplemental Fig. S1). Characteristics of the study cohort are presented in Table 1. More than half of the women in the study cohort were aged 45–55 years, 78% had ER+ tumors, 11% had triple-negative tumors, 56% were stage II, and 60% had breast-conserving surgery followed by intention-to-treat radiotherapy. One in ten patients had at least one prevalent comorbidity at the time of breast cancer diagnosis. Overall, 21 SNPs were successfully genotyped with call rates  ≥ 95% and five SNPs were excluded because of low call rates (*ABCB1* rs10248420, *CYP1A1* rs1048943, *TRPV1* rs879207, *ARHGEF10* rs9657362, and *EPHA8* rs209709). Information on call rates is provided in Table 2. For all included SNPs, the observed minor allele frequencies were consistent with those reported for European non-Finnish female populations, and the observed genotype frequencies were similar to the expected frequencies predicted under HWE (Table 2).

**Table 1:** Study cohort characteristics

	N	%
Total	2262	100
Age at diagnosis		
< 35	163	7.2
35–44	846	37.4
45–55	1253	55.4
ER status		
ER-	494	21.8
ER+	1768	78.2
HER-2 status		
Negative	1694	74.9
Positive	414	18.3
Not tested	154	6.8
Triple negative		
No	1918	84.8
Yes	249	11.0
Not tested	95	4.2
Positive lymph nodes		
None	893	39.5
1–2	987	43.6
3 or more	373	16.5
Missing	9	0.4
Tumor size (mm)		
≤ 20	≤ 1230	
21–50	964	42.6
51 or above	76	3.4
Missing	≤ 5	
TNM stage^a^		
Stage	567	25.1
Stage II	1285	56.8
Stage III	395	17.5
Missing	15	0.7
Pathological grade^b^		
Grade 1	343	15.2
Grade 2	934	41.3
Grade 3	744	32.9
Not graded	213	9.4
Missing	28	1.2
Comorbidity		
None	2028	89.7
1–2	162	7.2
3 or more	72	3.2
Surgery type	892	39.4
Mastectomy		
Breast-conserving surgery^c^	≤ 1375	
Missing	≤5	

*CCI* Charlson Comorbidity Index; *ER* estrogen receptor; *HER2* human epidermal growth factor 2; *TNBC* triple-negative breast cancer; *TNM* tumor, node, metastasis. In accordance with Danish law, data from cell sizes less than 5 and cells permitting back calculation are reported in aggregate

^a^Derived from tumor size and lymph node status

^b^Ductal and lobular tumors. Other tumors were not graded

^c^Including intention-to-treat radiotherapy

**Table 2:** Allele frequencies and associated genetic information

Gene	Ref SNP number	Benchmark MAF^b^	TaqMan assay ID	Call rate	Minor allele	Frequency	Observed				Expected			HWE Chi^2^	Excluded^c^
							Wildtype	Heterozygote	Homozygote	N/A	Wildtype	Heterozygote	Homozygote		
*ABCB1* ^a^	rs10248420	16%	C__30375194_10	64%	G	2%	3098	129	4	1856	3096	134	1	4.67	Yes
	rs1045642	47%	C___7586657_20	98%	G	45%	1508	2526	966	87	1536	2471	994	2.51	
	rs1128503	44%	C___7586662_10	97%	A	43%	1652	2371	930	134	1626	2424	904	2.36	
	rs2032582	46%	C_11711720C_30	96%	A	45%	1485	2373	1011	218	1466	2411	992	1.24	
*ABCC2*	rs12762549	48%	C__11214917_10	97%	G	46%	733	1138	544	69	702	1200	513	6.47	
*ABCG2*	rs2231142	11%	C__15854163_70	98%	A	10%	1961	446	25	53	1961	445	25	0.00	
*CYP1A1* ^a^	rs1048943	4%	C__25624888_50	39%	C	1%	1972	20	10	3085	1962	40	0	490.45	Yes
*CYP1B1*	rs1056836	43%	C___3099976_30	97%	C	44%	812	1093	508	72	461	1187	765	15.24	
*CYP3A* ^a^	rs10273424	9%	C__29554473_10	95%	A	8%	4076	748	31	232	4079	742	34	0.27	
*CYP3A4*	rs2740574	4%	C___1837671_50	97%	G	4%	2231	177	12	64	2223	193	4	15.98	
	rs35599367	5%	C__59013445_10	97%	T	3%	2251	144	8	82	2246	155	3	11.44	
*CYP3A5* ^a^	rs776746	7%	C__26201809_30	100%	T	8%	4335	700	37	15	4328	715	30	2.22	
*GSTP1*	rs1138272	9%	C___1049615_20	99%	T	8%	2090	352	19	24	2086	359	15	0.96	
*SLCO1B1*	rs2306283	40%	C_1901697_20	96%	C	41%	860	1095	432	98	830	1155	402	6.47	
	rs4149056	16%	C__30633906_10	96%	C	14%	1771	570	55	89	1764	584	48	1.28	
*SLCO1B3*	rs11045585	14%	C__31106434_10	98%	G	14%	1826	559	59	51	1814	583	47	4.22	
*ARHGEF10*	rs9657362	14%	C__25632922_10	90%	C	–	–	–	–	–	–	–	–	–	Yes
*EPHA4*	rs17348202	6%	C__34414779_10	98%	C	5%	2205	223	12	45	2199	234	6	1.28	
*EPHA5*	rs7349683	35%	C___1336545_30	99%	T	35%	1025	1116	307	37	1024	1118	305	0.01	
*EPHA6*	rs301927	16%	C___1037994_10	98%	G	17%	1665	673	86	61	1653	698	74	3.04	
*EPHA8*	rs209709	15%	C____702337_10	93%	G	–	–	–	–	–	–	–	–	–	Yes
*ERCC1*	rs11615	37%	C___2532959_1_	99%	G	37%	1118	991	341	35	1139	981	330	0.82	
	rs3212986	25%	C_2532948_10	98%	A	23%	1441	869	121	54	1447	857	127	0.47	
*ERCC2*	rs13181	38%	C___3145033_10	97%	G	37%	1008	1025	371	81	962	1118	325	16.51	
*FGD4*	rs10771973	30%	C__30728517_30	99%	A	29%	1249	991	208	37	1243	1003	202	0.33	
*TRPV1*	rs879207	32%	C___1497993_10	93%	G	32%	1131	880	301	173	1067	1007	237	36.78	Yes

^a^Genotyped in previous study

^b^Minor allele frequencies in the non-Finnish European female population

^c^Excluded due to low call rates. Clusters overlapped in rs10248420, rs1048943, rs9657362, and rs209709 genotyping, rendering manual adjustment unfeasible

During 10 years of follow-up (median: 7.0 years, IQR: 5.8–8.4 years), 249 women in our study cohort experienced a recurrence (cumulative incidence: 13.2%, 95% CI 11.5–15.0%). During 13 years of follow-up (median 10.1, IQR: 8.9–11.5), we observed 259 deaths (cumulative incidence: 16.0%, 95% CI 11.9–11.5%). Of these, 226 deaths were attributed to breast cancer (cumulative incidence: 12.6%, 95% CI 9.5–16.1%). Among the 33 deaths from other causes, 16 women died from other cancers, 11 died from other organ diseases or suicide, and six deaths were from unspecified causes.

**Fig. 1:**
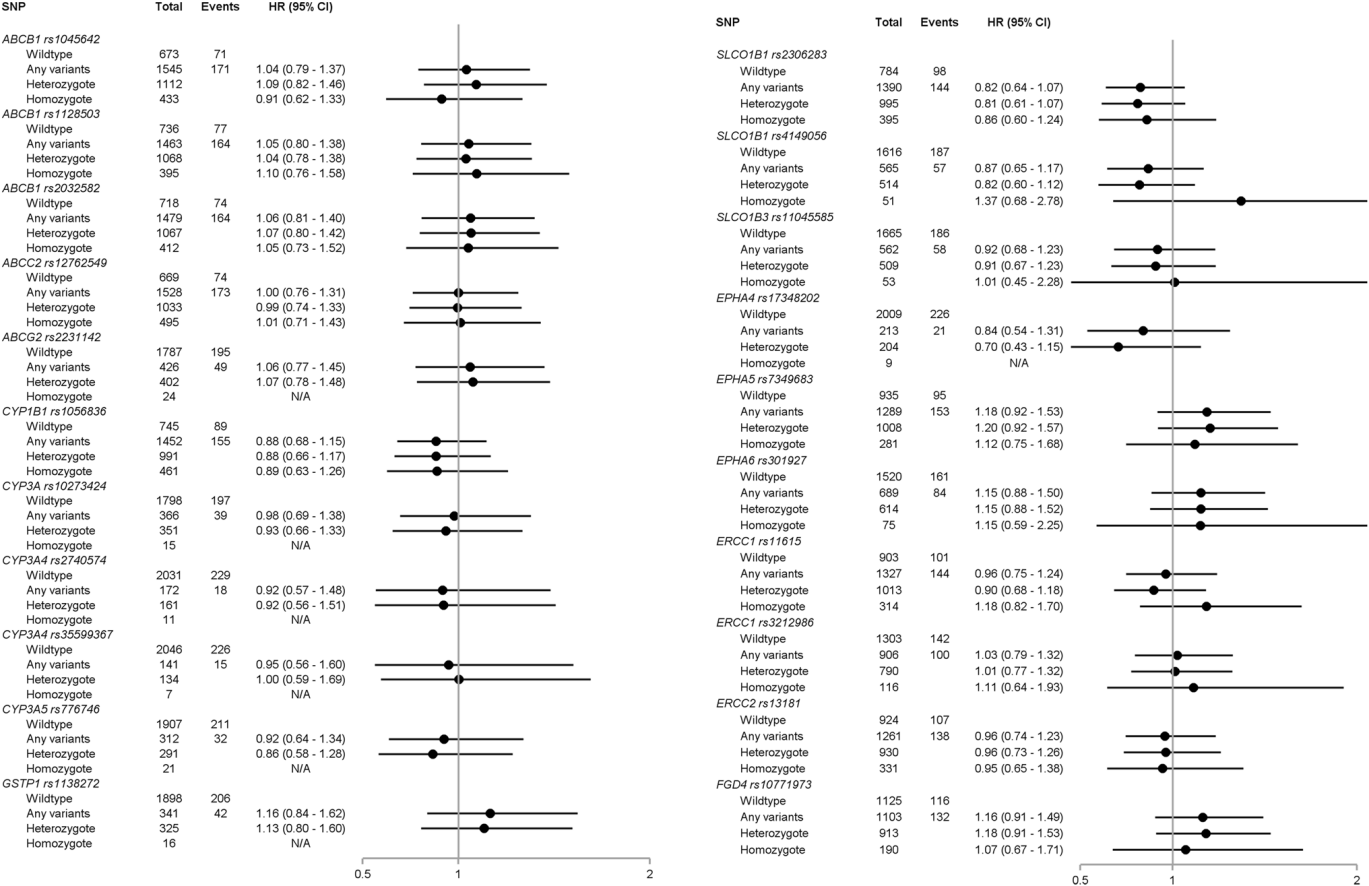
Hazard ratios and 95% CIs of breast cancer recurrence by SNPs

**Fig. 2:**
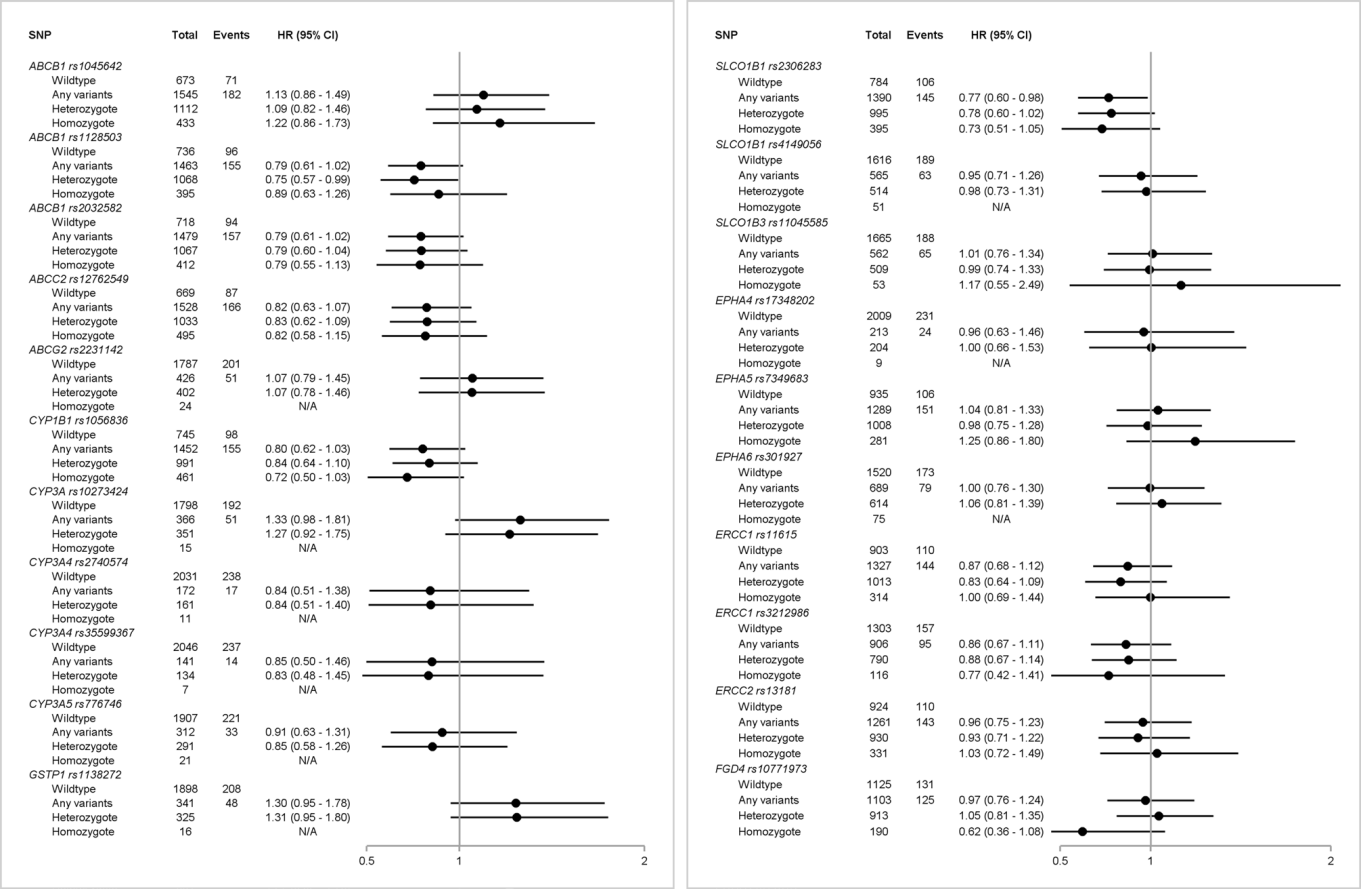
Hazard ratios and 95% CIs of all-cause mortality by SNPs

Figures 1 and 2 present SNP frequencies with HRs (of breast cancer recurrence and mortality, respectively). *SLCO1B1* rs2306283 was weakly associated with a reduced rate of recurrence (HR: 0.82, 95% CI 0.64–1.07) and mortality (HR: 0.77, 95% CI 0.60–0.98). We also observed minor associations between *GSTP1* rs1138272 and recurrence (HR: 1.16, 95% CI 0.84–1.62) and mortality (HR: 1.30, 95% CI 0.95–1.78). In contrast, we observed decreased recurrence (HR: 0.88, 95% CI 0.68–1.15) and mortality (HR: 0.80, 95% CI 0.62–1.03) among *CYP1B1* rs1056836 variant carriers. For some SNPs, we observed associations with mortality, but not recurrence. Mortality was decreased in variant carriers of the ABC transporters *ABCB1* rs1128503 (HR: 0.79, 95% CI 0.61–1.02), *ABCB1* rs2032582 (HR: 0.79, 95% CI 0.61–1.02), and *ABCC2* rs12762549 (HR: 0.82, 95% CI 0.63–1.07). In contrast, we observed increased mortality in variant carriers of *CYP3A* rs10273424 (HR: 1.33, 95% CI: 0.98–1.81). Analysis of SNPs encoding DNA repair genes suggested lower mortality in variant carriers of *ERCC1* rs11615 (HR: 0.87, 95% CI 0.68–1.12) and rs3212986 (HR: 0.86, 95% CI 0.67–1.11). We observed similar findings when we distinguished homozygote and heterozygote variant allele carriers.

When we pooled recurrences and BCSM as a single outcome, the HR slightly increased in variant carriers of *GSTP1* rs1138272 (HR: 1.23, 95% CI 0.92–1.64). In general, the estimates were similar in the analyses of all-cause mortality and BCSM, although the HR decreased for *SLCO1B1* rs2306283 (HR: 0.71, 95% CI 0.55–0.93) (Supplemental Tables S2–S3). Our findings were similar when we stratified by ER status and stage (Supplemental Tables S4–S5).

## Discussion

In this cohort of premenopausal women with non-distant metastatic breast cancer, carriers of the *SLCO1B1* rs2306283 variant had decreased recurrence and mortality rates compared with wildtypes. In contrast, we found increased mortality rates in *GSTP1* rs1138272 variant and *CYP3A* rs10273424 variant carriers. We did not observe any effect measure modification by ER status and stage.

Reduced function alleles of *SLCO1B1* are associated with docetaxel clearance in mice [30] but have not been associated with altered docetaxel clearance in humans [9, 30]. Longer systemic *paclitaxel* exposure has been observed among breast cancer patients carrying the rs4149056 and rs2306283 variants [31]. In a similar fashion, the apparent increased effectiveness of docetaxel that we observed in women with the rs2306283 variant may be attributable to reduced function of OATP1B1 and associated increased docetaxel plasma concentrations.

Polymorphisms in CYPs most often confer decreased enzyme activity [32, 33]. Theoretically, this would lead to increased docetaxel exposure and higher clinical efficacy. Yet our results suggested increased mortality, which also has been reported for other *CYP3A4* SNPs [14]. Currently, we do not have an explanation for these unexpected findings. Because *CYP3A* rs10273424 is an intronic variant, it could exert a causal effect via modified splicing or linkage disequilibrium. We considered it relevant to include in the current study as it has been associated with lower estrogen levels and increased breast cancer risk in premenopausal women [34].

Our observed decreased risk of recurrence and mortality among carriers of *CYP1B1* rs1056836 is consistent with some published research [35]. A recent study of 76 women (51% premenopausal) with triple-negative breast cancer undergoing adjuvant taxane, doxorubicin, and cyclophosphamide therapy found higher risk of recurrence in wildtypes (HR: 2.5, 95% CI 1.10–5.66), compatible with the favorable impact of variants observed in our study [35]. In a study of 58 Indian patients with advanced breast cancer (mixed pre- and postmenopausal), Tulsyan et al. [36] reported an association between *CYP3A5* rs776746 and complete or partial response to neo-adjuvant taxane treatment. We did not detect any associations between rs776746 and clinical outcomes in the adjuvant setting. GSTP1 overexpression has been associated with lower breast tumor reduction among patients treated with neo-adjuvant docetaxel or paclitaxel [37]. The mechanisms are yet unclear but could be reduced enzyme activity of GSTP1. Some studies report no associations between *GSTP1* polymorphisms and taxane-induced neuropathies [38, 39] and a meta-analysis by Ma et al*.* [40] found no association between *GSTP1* polymorphisms and breast cancer tumor response or overall survival. These studies were likely underpowered. In a Danish trial including 150 women with breast cancer *GSTP1* rs1138272 has been associated with docetaxel-induced peripheral neuropathy [41]. Whether and how this relates to our findings of increased recurrence and mortality are unclear, as this theoretically points toward increased the drug exposure.

A meta-analysis by Chen et al. [12], including prospective studies of taxane-treated breast, lung, ovarian, gastric, and head/neck cancer patients, reported better survival in *ABCB1* rs1128503 variant carriers, consistent with our findings [12]. Another meta-analysis including case–control studies did not detect such associations in breast cancer patients, but had limited precision [42]. In the study by Chen et al. [12], no overall associations were found for *ABCB1* rs1045642, but poor overall survival was found in European populations homozygote for the variant allele, consistent with the increased mortality observed in our study.

In other cancers, the *ERCC1* rs11625 wildtype and heterozygotes have been linked with toxicities after oxaliplatin-based chemotherapy [43], and similar to our findings, improved docetaxel effectiveness has been observed in variant carriers [44]. The previous studies were limited by small sample size (fewer than 62 patients), precluding definitive conclusions on the association of *ERCC1* SNPs and treatment effectiveness.

Our study has several noteworthy strengths. We examined the association between SNPs and docetaxel effectiveness in a population-based cohort of exclusively premenopausal women, included registry data with high validity and completeness [45], and systematically archived tumor tissue. Tumor tissue was collected at the time of primary breast cancer-directed surgery, thereby avoiding left truncation, selection, and immortal time bias. A key concern when using DNA extracted from FFPE tumors is whether the derived genotypes are representative of the germline, due to potential somatic genetic alterations. Previous studies show high genotype concordance between both FFPE breast tumors and FFPE normal lymph nodes [46] and between FFPE breast tumors and FFPE normal lymph nodes and whole blood [47]. We used tumor-infiltrated tissue, which has been suggested to be susceptible to genotyping error by loss of heterozygosity at certain genetic loci [48]. However, quantitative assessment of the influence of this in studies of *CYP2D6* rs3892097 showed minor impact of genotype misclassification when investigating breast cancer survival [47].

In four of the excluded SNPs (*ABCB1* rs10248420, *CYP1A1* rs1048943, *ARHGEF10* rs9657362, and *EPHA8* rs209709), genotype clusters overlapped widely, which could indicate poor performance of the assay in FFPE-extracted DNA. Hence, no manual adjustment was performed, and these SNPs were disregarded (along with one SNPs with call rates < 95%). This ensured high-quality genotyping data. We did not exclude SNPs with statistical evidence of departure from HWE. In studies with a large sample size, any deviation from HWE is likely to have minor practical importance [49, 50]. In the current study, congruity between the observed frequencies and those expected under HWE were reasonable, even in the SNPs departing from HWE.

Our study also had several limitations. First, we investigated the influence of single SNPs on taxane metabolism, precluding the evaluation of the synergistic effect of all SNPs. For example, the reduced activity of one ABC transporter might be offset by increased activity of other ABC family members. Second, we lacked detailed information on chemotherapy treatment, and while we know the women in our cohort received a minimum of one treatment cycle, dosing and information on chemotherapy type were not available. Instead, we restricted the cohort to the period when guideline treatment was docetaxel based and dosing was guided by body surface area. Third, we had no information on potential dose capping or early discontinuation, which may have been due to treatment-associated toxicities. Treatment modifications may be differentially distributed across genotypes and may hamper treatment benefits. Fourth, the associations observed in this study pertain to docetaxel administered *in combination with* cyclophosphamide and sometimes epirubicin, and not docetaxel monotherapy. Fifth, some SNPs were singled out based on findings from earlier studies that used other combinations of taxane-based chemotherapy [15, 35, 51–56]. As our study was based on routine clinical care data, our findings warrant confirmation in randomized clinical trials before genotyping can be used to guide taxane effectiveness in routine clinical practice.

Another concern is that despite the high validity and completeness of the DBCG clinical database, information on recurrences was not complete. A previous study reported a positive predictive value of 100% for recurrences in the DBCG, but a completeness of 70% using medical records as a reference standard [29]. We expected misclassification of recurrences to be non-differential across SNPs, and our sensitivity analysis pooling recurrence and BCSM suggested minor differential misclassification of recurrences, as the expanded analysis had little influence on two SNPs.

## Conclusion

This study, focusing on premenopausal non-distant metastatic breast cancer patients treated with taxane chemotherapy, demonstrated that some SNPs involved in docetaxel pharmacogenomics may impact breast cancer recurrence and/or mortality, especially *SLCO1B1* rs2306283, *GSTP1* rs1138272, and *CYP3A* rs10273424. Findings from this study merit further investigation. They will be used in future work using multiple pathway analysis [23, 57] to capture the net effect of these SNPs. Also, future clinical trials are needed to elucidate whether genomic testing could guide dosing of taxane-based therapy to reduce inter-individual variability in effectiveness.

## Supplementary Information

Below is the link to the electronic supplementary material.

Supplementary file1 (DOCX 790 kb)

## Declarations

### Data availability

The data used for the current study derive from nationwide, population-based administrative and medical registries, linked anonymously using a personalized identifier. The data are available from Statistics Denmark, DBCG and the corresponding author. Restrictions apply to the availability of these data, which were used under license for this study. Data are available from the authors upon reasonable request with the permission of Statistics Denmark, DBCG, The Danish Health Authorities, The Danish Data Protection Agency, and The Central Jutland Region Committee on Health Research Ethics.
