# Supplementary material for: Single-nucleotide polymorphisms and the effectiveness of taxane-based chemotherapy in premenopausal breast cancer: a population-based cohort study in Denmark
Source: Breast Cancer Res Treat. 2022 Apr 30;194(2):353–63. doi: 10.1007/s10549-022-06596-2 (PMC9239972; doi:10.1007/s10549-022-06596-2)
Supplement: Supplementary file 1 — Supplementary file1 (DOCX 790 kb) [file 10549_2022_6596_MOESM1_ESM.docx]

**Supplemental material**

Single nucleotide polymorphisms and the effectiveness of taxane-based chemotherapy in premenopausal breast cancer:

A population-based cohort study in Denmark

**Contents**

[Figure S1. Flow diagram, study cohort 2](#_Toc93909594)

[Table S1. Algorithm for Charlson Comorbidity Index 3](#_Toc93909595)

[Table S2. Associations between SNPs and recurrence or BCSM 4](#_Toc93909596)

[Table S3. Associations between SNPs and BCSM 5](#_Toc93909597)

[Table S4. Associations between SNPs and recurrence and mortality by ER status 6](#_Toc93909599)

[Table S5. Associations between SNPs and recurrence and mortality by stage 7](#_Toc93909600)

# Figure S1. Flow diagram, study cohort.

| All women in the ProBe CaRe cohort  n=5,959 | | | | | | |  |
| --- | --- | --- | --- | --- | --- | --- | --- |
|  |  |  | |  | | | Excluded |
|  |  |  | |  | | | Women diagnosed before 2007  n=2980 |
|  |  |  | |  | | |  |
|  |  |  | |  | | |  |
|  | |  | | | | | Women aged >55 years  n=30 |
|  |  |  |  | |  | |  |
|  |  |  |  | | |  |  |
|  |  |  |  | | |  | Women who did not receive chemotherapy^a^  n=314 |
|  |  |  |  | | |  |  |
|  |  |  | |  | | |  |
|  |  |  | |  | | | Women with no available tumor tissue  n=355 |
|  |  |  | |  | | |  |
|  |  |  | |  | | |  |
|  |  |  | |  | | | End of follow-up within 6 months  n=18 |
|  |  |  | |  | | |  |
|  |  |  | |  | | |  |
| Study cohort  Premenopausal women diagnosed with breast cancer 2007-2011 assigned adjuvant chemotherapy  n=2262 | | | | | | |  |

The study cohort was nested in the ProBe CaRe cohort. During establishment of the ProBe CaRe cohort, ER+ patients not treated with tamoxifen, ER– patients treated with tamoxifen, and women with missing information on ER status or tamoxifen were excluded (303 women diagnosed after 2007). Women listed as premenopausal at primary diagnosis who were older than 55 years were excluded. We also excluded women diagnosed before 2007 and women who did not receive chemotherapy.

^a^ Tumor characteristics differed in women not receiving chemotherapy, compared to women treated with chemotherapy. The latter had higher proportions of stage l tumors (61% vs. 26%), grade 1 tumors (34% vs. 15%), ER+ status (83% vs. 78%), and women aged 45-55 (63% vs. 55%).

^b^ Due to recurrence (n≤15), other malignancies, or death (n≤5).

# Table S1. Algorithm for Charlson Comorbidity Index according to codes in the *International Classification of Diseases, Tenth Revision.*

|  | **Diseases** | **ICD-10** | **Score** |
| --- | --- | --- | --- |
| 1 | Myocardial infarction | I21;I22;I23 | 1 |
| 2 | Congestive heart failure | I50; I11.0; I13.0; I13.2 | 1 |
| 3 | Peripheral vascular disease | I70; I71; I72; I73; I74; I77 | 1 |
| 4 | Cerebrovascular disease | I60-I69; G45; G46 | 1 |
| 5 | Dementia | F00-F03; F05.1; G30 | 1 |
| 6 | Chronic pulmonary disease | J40-J47; J60-J67; J68.4; J70.1; J70.3; J84.1; J92.0; J96.1; J98.2; J98.3 | 1 |
| 7 | Connective tissue disease | M05; M06; M08; M09; M30; M31; M32; M33; M34; M35; M36; D86 | 1 |
| 8 | Ulcer disease | K22.1; K25-K28 | 1 |
| 9 | Mild liver disease | B18; K70.0-K70.3; K70.9; K71; K73; K74; K76.0 | 1 |
| 10 | Diabetes type1  Diabetes type2 | E10.0, E10.1; E10.9  E11.0; E11.1; E11.9 | 1 |
| 11 | Hemiplegia | G81; G82 | 2 |
| 12 | Moderate to severe renal disease | I12; I13; N00-N05; N07; N11; N14; N17-N19; Q61 | 2 |
| 13 | Diabetes with end-organ damage type1 and type2 | E10.2-E10.8  E11.2-E11.8 | 2 |
| 14 | Any tumor (except BC) | C00-C75 (excluding C50) | 2 |
| 15 | Leukemia | C91-C95 | 2 |
| 16 | Lymphoma | C81-C85; C88; C90; C96 | 2 |
| 17 | Moderate to severe liver disease | B15.0; B16.0; B16.2; B19.0; K70.4; K72; K76.6; I85 | 3 |
| 18 | Metastatic solid tumor | C76-C80 | 6 |
| 19 | AIDS | B21-B24 | 6 |

The Danish National Patient Registry covers all Danish Hospitals and has registered data on all non-psychiatric inpatient admissions and outpatient visits since 1977 [1].

Reference:

1. Schmidt M, Schmidt SAJ, Sandegaard JL, Ehrenstein V, Pedersen L, Sørensen HT. The Danish National Patient Registry: a review of content, data quality, and research potential. Clin Epidemiol. 2015;7:449–90.

# Table S2. Associations between SNPs and recurrence or BCSM.


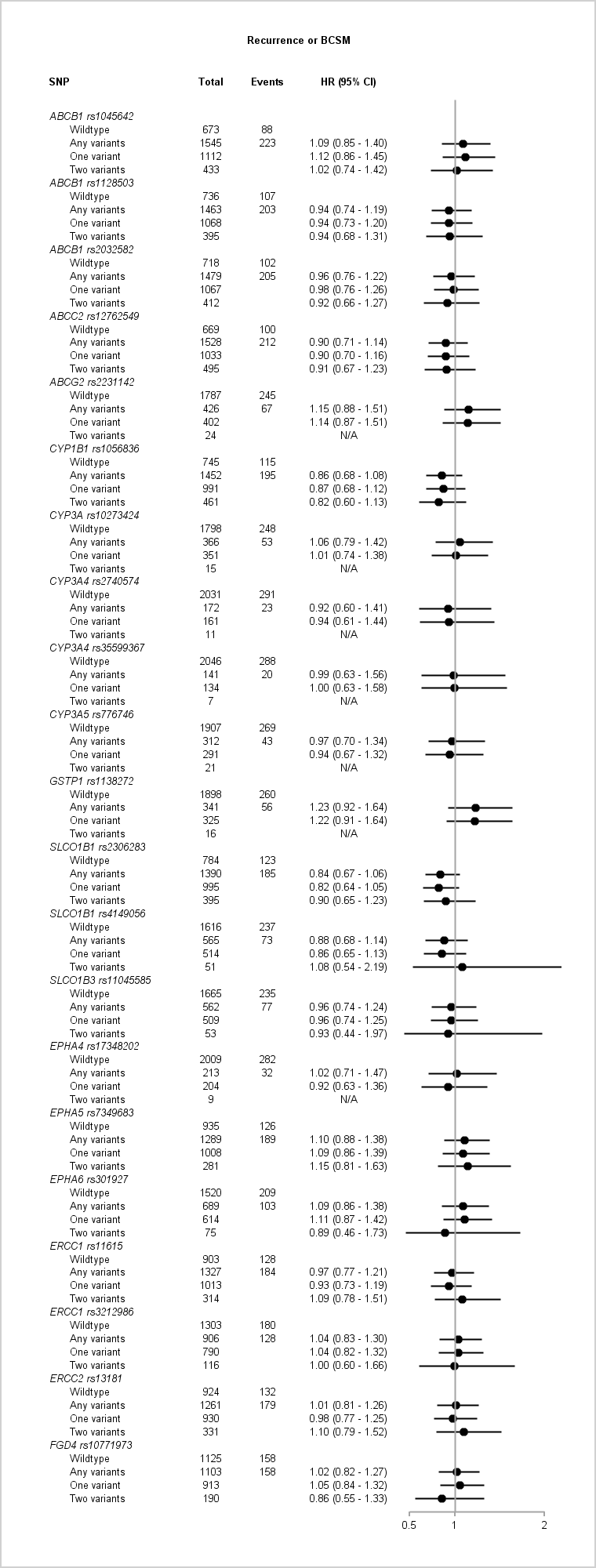


# Table S3. Associations between SNPs and BCSM.


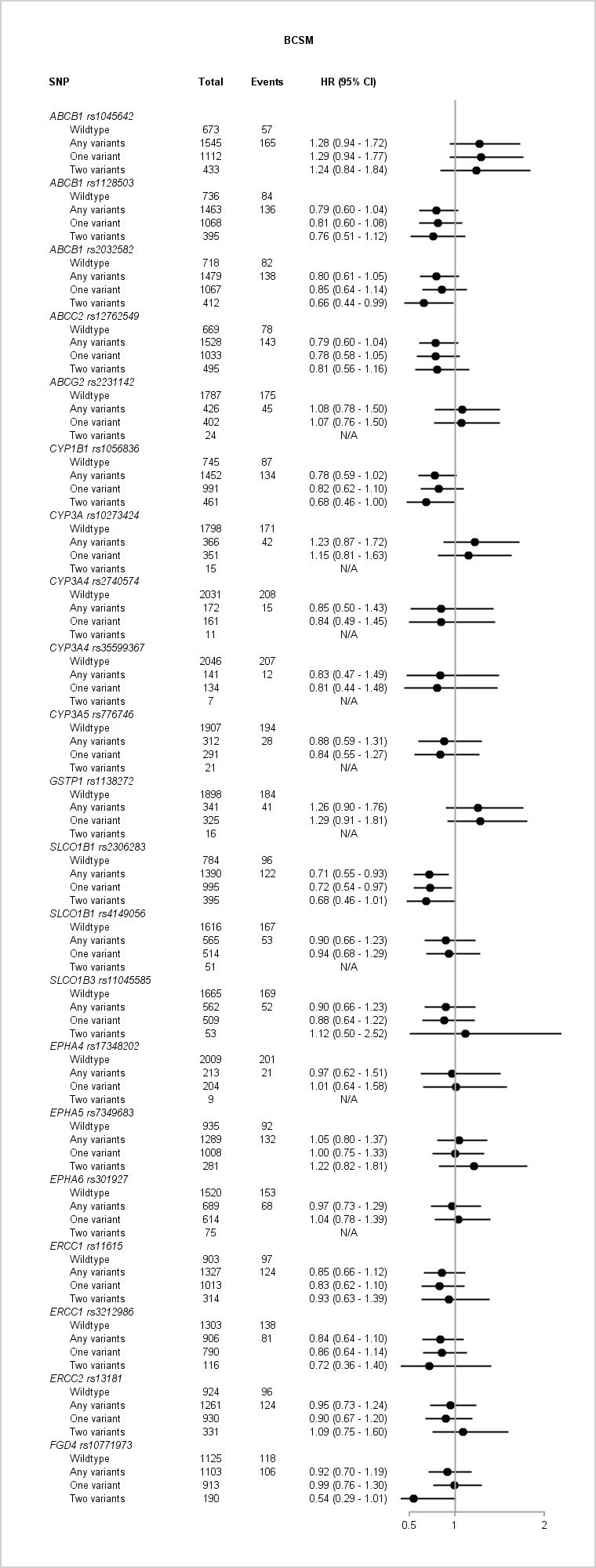


# Table S4. Associations between SNPs and recurrence and mortality by ER status.

|  | **Recurrence** | | **Mortality** | |
| --- | --- | --- | --- | --- |
| SNP | ER+ | ER- | ER+ | ER- |
| ***ABCB1* rs1045642** |  |  |  |  |
| Any variants | 0.96 (0.70 - 1.32) | 1.26 (0.72 - 2.18) | 0.98 (0.71 - 1.37) | 1.41 (0.85 - 2.34) |
| ***ABCB1* rs1128503** |  |  |  |  |
| Any variants | 0.98 (0.72 - 1.35) | 1.24 (0.74 - 2.10) | 0.75 (0.55 - 1.02) | 0.88 (0.57 - 1.36) |
| ***ABCB1* rs2032582** |  |  |  |  |
| Any variants | 1.01 (0.73 - 1.40) | 1.22 (0.72 - 2.06) | 0.74 (0.54 - 1.01) | 0.93 (0.60 - 1.44) |
| ***ABCC2* rs12762549** |  |  |  |  |
| Any variants | 1.12 (0.81 - 1.55) | 0.72 (0.44 - 1.17) | 0.88 (0.64 - 1.22) | 0.67 (0.44 - 1.03) |
| ***ABCG2* rs2231142** |  |  |  |  |
| Any variants | 1.09 (0.75 - 1.57) | 1.00 (0.56 - 1.80) | 1.06 (0.72 - 1.56) | 1.10 (0.66 - 1.82) |
| ***CYP1B1* rs1056836** |  |  |  |  |
| Any variants | 0.94 (0.69 - 1.28) | 0.77 (0.48 - 1.23) | 0.89 (0.65 - 1.23) | 0.65 (0.42 - 0.98) |
| ***CYP3A* rs10273424** |  |  |  |  |
| Any variants | 0.90 (0.59 - 1.36) | 1.18 (0.64 - 2.15) | 1.26 (0.85 - 1.85) | 1.40 (0.84 - 2.34) |
| ***CYP3A4* rs2740574** |  |  |  |  |
| Any variants | 1.07 (0.62 - 1.83) | 0.57 (0.21 - 1.53) | 0.97 (0.54 - 1.75) | 0.58 (0.24 - 1.44) |
| ***CYP3A4* rs35599367** |  |  |  |  |
| Any variants | 0.90 (0.48 - 1.68) | 1.02 (0.40 - 2.60) | 0.46 (0.19 - 1.12) | 1.50 (0.75 - 3.00) |
| ***CYP3A5* rs776746** |  |  |  |  |
| Any variants | 1.07 (0.70 - 1.62) | 0.59 (0.26 - 1.37) | 0.87 (0.55 - 1.37) | 1.00 (0.55 - 1.85) |
| ***GSTP1* rs1138272** |  |  |  |  |
| Any variants | 1.40 (0.96 - 2.04) | 0.66 (0.33 - 1.33) | 1.27 (0.85 - 1.89) | 1.25 (0.75 - 2.08) |
| ***SLCO1B1* rs2306283** |  |  |  |  |
| Any variants | 0.84 (0.62 - 1.14) | 0.70 (0.43 - 1.13) | 0.68 (0.50 - 0.92) | 0.88 (0.56 - 1.37) |
| ***SLCO1B1* rs4149056** |  |  |  |  |
| Any variants | 0.83 (0.58 - 1.19) | 0.93 (0.54 - 1.57) | 0.88 (0.61 - 1.26) | 1.02 (0.64 - 1.63) |
| ***SLCO1B3* rs11045585** |  |  |  |  |
| Any variants | 1.05 (0.75 - 1.47) | 0.63 (0.34 - 1.16) | 1.16 (0.82 - 1.62) | 0.80 (0.47 - 1.33) |
| ***EPHA4* rs17348202** |  |  |  |  |
| Any variants | 0.95 (0.58 - 1.56) | 0.56 (0.21 - 1.51) | 1.12 (0.69 - 1.83) | 0.68 (0.30 - 1.56) |
| ***EPHA5* rs7349683** |  |  |  |  |
| Any variants | 1.06 (0.78 - 1.42) | 1.65 (1.00 - 2.73) | 1.07 (0.79 - 1.46) | 0.99 (0.65 - 1.51) |
| ***EPHA6* rs301927** |  |  |  |  |
| Any variants | 1.08 (0.78 - 1.47) | 1.27 (0.79 - 2.05) | 0.96 (0.69 - 1.34) | 0.98 (0.63 - 1.52) |
| ***ERCC1* rs11615** |  |  |  |  |
| Any variants | 0.98 (0.73 - 1.33) | 0.90 (0.56 - 1.45) | 0.86 (0.63 - 1.17) | 0.90 (0.59 - 1.37) |
| ***ERCC1* rs3212986** |  |  |  |  |
| Any variants | 0.92 (0.68 - 1.25) | 1.38 (0.86 - 2.21) | 0.83 (0.60 - 1.13) | 1.00 (0.65 - 1.54) |
| ***ERCC2* rs13181** |  |  |  |  |
| Any variants | 1.00 (0.74 - 1.34) | 0.92 (0.58 - 1.46) | 1.00 (0.73 - 1.36) | 0.95 (0.62 - 1.44) |
| ***FGD4* rs10771973** |  |  |  |  |
| Any variants | 1.12 (0.84 - 1.51) | 1.19 (0.74 - 1.90) | 0.91 (0.67 - 1.24) | 0.99 (0.65 - 1.49) |

# Table S5. Associations between SNPs and recurrence and mortality by stage.

|  | **Recurrence** | | **Mortality** | |
| --- | --- | --- | --- | --- |
| SNP | Stage І-ІІ | Stage ІІІ | Stage І-ІІ | Stage ІІІ |
| ***ABCB1* rs1045642** |  |  |  |  |
| Any variants | 1.02 (0.72 - 1.45) | 0.92 (0.59 - 1.44) | 1.08 (0.76 - 1.52) | 1.05 (0.67 - 1.65) |
| ***ABCB1* rs1128503** |  |  |  |  |
| Any variants | 1.11 (0.78 - 1.57) | 1.07 (0.69 - 1.65) | 0.81 (0.58 - 1.12) | 0.80 (0.53 - 1.20) |
| ***ABCB1* rs2032582** |  |  |  |  |
| Any variants | 1.19 (0.83 - 1.71) | 0.99 (0.65 - 1.53) | 0.84 (0.60 - 1.17) | 0.77 (0.51 - 1.14) |
| ***ABCC2* rs12762549** |  |  |  |  |
| Any variants | 1.03 (0.72 - 1.46) | 1.02 (0.66 - 1.58) | 0.99 (0.70 - 1.40) | 0.65 (0.43 - 0.97) |
| ***ABCG2* rs2231142** |  |  |  |  |
| Any variants | 1.00 (0.67 - 1.50) | 1.21 (0.74 - 1.98) | 1.15 (0.78 - 1.69) | 0.96 (0.58 - 1.61) |
| ***CYP1B1* rs1056836** |  |  |  |  |
| Any variants | 1.03 (0.73 - 1.45) | 0.70 (0.47 - 1.05) | 0.82 (0.59 - 1.13) | 0.78 (0.52 - 1.16) |
| ***CYP3A* rs10273424** |  |  |  |  |
| Any variants | 0.77 (0.48 - 1.25) | 1.25 (0.76 - 2.04) | 1.21 (0.80 - 1.83) | 1.39 (0.87 - 2.22) |
| ***CYP3A4* rs2740574** |  |  |  |  |
| Any variants | 0.96 (0.54 - 1.72) | 1.01 (0.45 - 2.28) | 0.77 (0.41 - 1.47) | 1.18 (0.55 - 2.54) |
| ***CYP3A4* rs35599367** |  |  |  |  |
| Any variants | 0.98 (0.50 - 1.92) | 0.79 (0.36 - 1.78) | 1.06 (0.56 - 2.02) | 0.49 (0.18 - 1.33) |
| ***CYP3A5* rs776746** |  |  |  |  |
| Any variants | 0.89 (0.55 - 1.44) | 1.00 (0.55 - 1.79) | 0.69 (0.41 - 1.16) | 1.32 (0.79 - 2.23) |
| ***GSTP1* rs1138272** |  |  |  |  |
| Any variants | 1.14 (0.75 - 1.74) | 1.31 (0.76 - 2.26) | 1.43 (0.97 - 2.11) | 1.21 (0.71 - 2.07) |
| ***SLCO1B1* rs2306283** |  |  |  |  |
| Any variants | 0.88 (0.63 - 1.22) | 0.75 (0.50 - 1.14) | 0.82 (0.59 - 1.13) | 0.70 (0.47 - 1.05) |
| ***SLCO1B1* rs4149056** |  |  |  |  |
| Any variants | 1.01 (0.70 - 1.45) | 0.65 (0.39 - 1.11) | 0.99 (0.69 - 1.41) | 0.88 (0.55 - 1.40) |
| ***SLCO1B3* rs11045585** |  |  |  |  |
| Any variants | 1.08 (0.75 - 1.54) | 0.74 (0.44 - 1.24) | 1.10 (0.77 - 1.56) | 0.93 (0.58 - 1.50) |
| ***EPHA4* rs17348202** |  |  |  |  |
| Any variants | 0.56 (0.29 - 1.09) | 1.36 (0.77 - 2.41) | 0.98 (0.57 - 1.67) | 0.92 (0.46 - 1.83) |
| ***EPHA5* rs7349683** |  |  |  |  |
| Any variants | 1.08 (0.78 - 1.49) | 1.28 (0.83 - 1.97) | 0.92 (0.67 - 1.26) | 1.16 (0.77 - 1.75) |
| ***EPHA6* rs301927** |  |  |  |  |
| Any variants | 1.25 (0.90 - 1.74) | 1.10 (0.71 - 1.70) | 0.99 (0.70 - 1.39) | 1.06 (0.70 - 1.63) |
| ***ERCC1* rs11615** |  |  |  |  |
| Any variants | 0.93 (0.67 - 1.28) | 0.96 (0.63 - 1.46) | 0.87 (0.63 - 1.19) | 0.84 (0.57 - 1.26) |
| ***ERCC1* rs3212986** |  |  |  |  |
| Any variants | 1.08 (0.78 - 1.49) | 0.96 (0.63 - 1.46) | 0.90 (0.65 - 1.25) | 0.80 (0.53 - 1.20) |
| ***ERCC2* rs13181** |  |  |  |  |
| Any variants | 1.01 (0.73 - 1.39) | 0.97 (0.65 - 1.46) | 1.09 (0.79 - 1.51) | 0.83 (0.56 - 1.23) |
| ***FGD4* rs10771973** |  |  |  |  |
| Any variants | 1.28 (0.93 - 1.76) | 1.14 (0.76 - 1.70) | 1.02 (0.74 - 1.39) | 1.06 (0.72 - 1.57) |
